# Effects of the expansion of integrated maternal‐fetal intensive care centers on high‐risk newborns in Korea

**DOI:** 10.1002/ijgo.16085

**Published:** 2025-01-30

**Authors:** Ye Seul Jang, Sung‐In Jang, Eun‐Cheol Park

**Affiliations:** ^1^ Department of Public Health Graduate School, Yonsei University Seoul Republic of Korea; ^2^ Institute of Health Services Research Yonsei University Seoul Republic of Korea; ^3^ Department of Preventive Medicine Yonsei University College of Medicine Seoul Republic of Korea

**Keywords:** health policy, high‐risk birth, interrupted time series, low‐birth‐weight delivery, maternal‐fetal intensive care unit, preterm birth

## Abstract

**Objective:**

To investigate the impact of the introduction of Integrated Maternal‐Fetal Intensive Care Unit (MFICU) reimbursement rates for high‐risk newborns in South Korea.

**Method:**

The present study used data from the Population Dynamics data released annually by Statistics Korea, which contain information on all births in the country from October 1, 2015, to September 31, 2019. The MFICU reimbursement fee began on October 1, 2017, and the follow‐up period was 24 months before and after the intervention. The dependent variable was defined as either premature births (before 37 weeks of pregnancy) or underweight births (birth weight ≤2.5 kg). A total of 1 377 841 infants were included in the present study, and an Interrupted Time Series with segmented regression analysis was performed.

**Results:**

After the intervention, the premature or low‐birth‐weight deliveries decreased by approximately 0.4%. The difference in the level change was more significant in legitimate children and multiple births. Premature neonates showed a significantly different level of change compared with low‐birth‐weight neonates.

**Conclusion:**

The expansion of the MFICU reduces high‐risk births, such as premature births and those involving low birth weight. To effectively care for high‐risk deliveries, the enhancement of obstetric care and continuous medical support policies must be maintained.

## Synopsis

The introduction of reimbursement rates for maternal‐fetal intensive care in South Korea correlated with a decrease in premature and low‐birth‐weight deliveries by approximately 0.4%.

## 1 INTRODUCTION

In Korea, the age of first marriage is increasing annually. In 2022, the average age at first marriage for men and women was 33.7 and 31.3 years, respectively.^1^ This delay in age is associated with negative outcomes, such as advanced maternal age, infertility, and miscarriage. A systematic review indicated that advanced maternal age increases the risk of requiring obstetric interventions.^2^ According to a population trend survey by Statistics Korea, although the birth rate among those under 35 years of age is decreasing, the birth rate among those aged 35 years and older will increase by 1.2 percentage points to 35.0% in 2021, indicating an increase in the likelihood of high‐risk pregnancies.^3^

Preterm birth refers to the birth of a baby before 37 weeks of pregnancy. It is a significant global issue, accounting for an average of 5%–18% of births worldwide and a leading cause of death among children under 5 years old.^4^ High‐risk pregnancy typically refers to a condition in which the probability of maternal or fetal death or disease is higher than that in normal pregnancies or where complications before and after delivery are more likely to occur than in normal pregnancies.^5^ Pregnancy is a unique but physiologically normal event experienced by women throughout their reproductive years. However, a combination of pre‐existing or unexpected maternal conditions during pregnancy can increase the likelihood of adverse outcomes.^6^ Before pregnancy, all women may have biologic, psychological, social, and clinical risk factors for premature birth, depending on how they manage their health. Controllable risk factors can reduce the risk of adverse maternal outcomes through preventive management and active response strategies.^7^

Pregnant women require inpatient treatment in specialized and intensive care facilities to reduce the risk of complications during pregnancy and to detect such complications early.^8^, ^9^ In contrast, Easter et al.^10^ reported that approximately 68.2% of high‐risk pregnant women, such as those with a history of uterine surgery, gave birth in medical institutions that were without appropriate medical capabilities. Among these, deficiencies in comprehensive transportation systems have been identified as critical issues. underscoring the need for systematic improvements. This emphasizes the importance of specialized medical infrastructure for high‐risk pregnancies.

In Korea, the Ministry of Health and Welfare supports intensive treatment to ensure safe delivery in women with high‐risk pregnancies.^11^ The management of high‐risk pregnant women and newborns is systematically coordinated from the period before delivery of the high‐risk mother and fetus to the period after delivery of the high‐risk newborn. To this end, an emergency medical system is maintained, and the Integrated Maternal‐Fetal Intensive Care Unit (MFICU) plays a crucial role in this process. Since the second half of 2017, management and hospitalization fees for high‐risk pregnant women have been established to expand facilities for the voluntary treatment of high‐risk pregnant women. This reimbursement fee was introduced to cover the costs associated with the establishment and operation of High‐Risk Maternal and Neonatal Intensive Care Units (MFICUs). These specialized units are designed to simultaneously monitor and provide care for both the mother and fetus, particularly in high‐risk pregnancies. The reimbursement fee helps to cover the costs of the unique infrastructure, equipment, and medical personnel required to ensure optimal care for both the mother and neonate. Specifically, the fee covers the provision of separate, dedicated space within the MFICU, allowing for dual monitoring of maternal and fetal health, as well as the specialized medical equipment necessary for this purpose. Additionally, the reimbursement fee supports the staffing of pediatric specialists who must be on‐site in these units, as well as maintaining a nurse‐to‐bed ratio of no more than 1.5 beds per nurse, which is essential for providing adequate care. In cases where hospitals fail to meet these requirements, such as not having a separate space or failing to meet the required nurse‐to‐bed ratio, the reimbursement fee is adjusted accordingly and set at a lower rate. By introducing this fee, the government aims to ensure that hospitals are adequately supported in providing specialized care for high‐risk pregnancies while promoting access to advanced maternal and neonatal care across medical institutions. With the increasing number of high‐risk pregnant women and newborns and the decreasing birth rate, continuous attention to MFICU is necessary.^12^ However, there is a scarcity of research assessing the efficacy of MFICU following the introduction of fees. Hence, this study sought to investigate how the expansion of MFICU has influenced high‐risk deliveries, specifically premature births or low birth weight.

## 2 MATERIALS AND METHODS

### 2.1 Data and participants

In the present study, we analyzed birth‐related data from the Population Dynamics data provided by Statistics Korea through the Microdata Integration Service.^13^ Statistics Korea publishes monthly population trends, and we used these data to examine information on pregnant women, gestational age, birth weight at delivery, race, parity, total births, and sociodemographic characteristics of parents. The study protocol was approved by the Institutional Review Board of Severance Hospital, Yonsei University Health System. The requirement for informed consent was waived because the Causes of Death Statistics did not contain any identifiable information. Furthermore, ethical approval for the use of the data was not required because Statistics Korea provides publicly accessible data.

The present study included data spanning 48 months, divided into 24 months before and after October 1, 2017, when reimbursement rates for integrated MFICU were introduced in South Korea. All newborns born during this period were included, totaling 1 377 841 infants, after excluding those with missing sociodemographic data.

### 2.2 Variables

The main variable of interest was the Ministry of Health and Welfare of Korea, which implemented the MFICU‐related reimbursement fee on October 1, 2017. The pre‐intervention period was from October 1, 2015, to September 30, 2017, and the post‐intervention period was from October 1, 2017, to September 30, 2019.

The dependent variable was defined as the case of satisfaction with either preterm or underweight births. Preterm infants refer to infants born before 37 weeks of pregnancy, which is generally used as the cut‐off to distinguish preterm births. Specifically, this category includes infants born before 37 weeks of pregnancy.^14^ On the other hand, low birth weight refers to infants whose birth weight is 2.5 kg or less at birth, indicating interrupted or delayed normal growth until delivery, regardless of gestational age.^15^ All variables were based on birth registration.

Covariates included sociodemographic factors such as sex of the baby (male and female), parents' age at birth (≤19, 20–29, 30–39, 40–49, 50 years and older), history of birth, place of delivery, multiple‐fetal (single, multiple), residential areas (medically vulnerable or not), parents' education level (low [below elementary school]), middle [under high school graduation], high [above university level], marital status (married and single) occupation of parents (white collar [professional, managerial, or administrative workers]), blue collar [passive or industrial workers], pink collar [service oriented workers], and race (Korean, non‐Korean).

### 2.3 Statistical analysis

Descriptive statistics were presented as frequencies and percentages. To investigate and compare the general characteristics of the study population, a *χ* ^2^ test was conducted. Subsequently, an interrupted time series with segmented regression was performed to analyze the time trends and changes in outcomes. The interrupted time series is modeled using a linear regression model, including three time‐related variables, and the regression coefficients estimate the pre‐intervention slope, level change at the time of the intervention, and post‐intervention slope change. Slope change quantified the difference between the pre‐ and post‐intervention slopes. The level change represents an absolute change in the level of outcomes at the time of the intervention and measures the immediate effect of the intervention.^16^ Because we applied the logit‐link function to the generalized linear model to perform the segmented regression, the model coefficients were converted into exponentials to represent the trends and changes in the outcomes on the original scale. To interpret the model coefficients, log[E(Yi)] needed to be converted into multiplicative interpretations for the original scale E(Yi) = μi: log(μi) = *β*0 + *β*1 × time t + *β*2 × intervention t + *β*3 × time after intervention t + et.

In this model, the intercept *β*0 estimates the baseline level of the outcome; *β*1 estimates the baseline trend of the outcome; *β*2 estimates the level change after the intervention, which indicates the immediate effect size of the intervention; *β*3 estimates the change in trend after the intervention; and the sum of *β*1 and *β*3 is the slope after the intervention, indicating the follow‐up outcome trend.^17^ Parameter estimates, standard errors, and *P* values are presented as key results. SAS version 9.4 statistical software (SAS Institute Inc., Cary, NC, USA) was used for all analyses. Statistical significance was set at a two‐sided value of *P* less than 0.05.

## 3 RESULTS

A total of 1 377 841 individuals were included in the analysis. The total number of births before the introduction of the intervention (*n* = 759 533) decreased (*n* = 618 308). Legitimate children (1 363 235; 98.9%), mothers in their 30s (985 995; 71.5%), fathers in their 30s (1 029 812; 74.7%), those who live in medically served areas (1 353 467; 98.2%), and those who had a delivery in a hospital (1 370 761; 99.4%) were most frequently reported among all participants. Totals of 71 112 and 64 194 births were classified as high‐risk newborns before and after the intervention, respectively (Table 1).

**TABLE 1:** Characteristics of the study population.^a^

Variables	High‐risk newborn (*n* = 1 377 841)									
	Pre‐intervention					Post‐intervention				
	Total (*n* = 759 533)	High‐risk newborn (*n* = 71 112)				Total (*n* = 618 308)	High‐risk newborn (*n* = 64 194)			
		No	%	Yes	%		No	%	Yes	%
Sex										
Male	389 871	353 186	(90.59)	36 685	(9.41)	317 647	284 487	(89.56)	33 160	(10.44)
Female	369 662	335 235	(90.69)	34 427	(9.31)	300 661	269 627	(89.68)	31 034	(10.32)
Legitimate child										
Yes	7150	6287	(87.93)	863	(12.07)	7456	6439	(86.36)	1017	(13.64)
No	752 383	682 134	(90.66)	70 249	(9.34)	610 852	547 675	(89.66)	63 177	(10.34)
Mother's age, y										
≤19	2368	2093	(88.39)	275	(11.61)	1594	1418	(88.96)	176	(11.04)
20–29	193 215	178 022	(92.14)	15 193	(7.86)	145 260	133 150	(91.66)	12 110	(8.34)
30–39	539 750	487 113	(90.25)	52 637	(9.75)	446 245	397 898	(89.17)	48 347	(10.83)
40–49	24 180	21 182	(87.60)	2998	(12.40)	25 181	21 639	(85.93)	3542	(14.07)
50–59	20	11	(55.00)	9	(45.00)	28	9	(32.14)	19	(67.86)
Father's age, y										
≤19	857	750	(87.51)	107	(12.49)	442	386	(87.33)	56	(12.67)
20–29	88 665	81 553	(91.98)	7112	(8.02)	67 146	61 441	(91.50)	5705	(8.50)
30–39	571 300	518 605	(90.78)	52 695	(9.22)	458 512	412 101	(89.88)	46 411	(10.12)
40–49	95 483	84 720	(88.73)	10 763	(11.27)	88 350	76 875	(87.01)	11 475	(12.99)
50–59	3228	2793	(86.52)	435	(13.48)	3858	3311	(85.82)	547	(14.18)
Mother's education level										
Below elementary school	1852	1639	(88.50)	213	(11.50)	1523	1352	(88.77)	171	(11.23)
Middle to high school	164 736	147 473	(89.52)	17 263	(10.48)	122 154	108 263	(88.63)	13 891	(11.37)
Above university level	592 945	539 309	(90.95)	53 636	(9.05)	494 631	444 499	(89.86)	50 132	(10.14)
Father's education level										
Below elementary school	1561	1395	(89.37)	166	(10.63)	1157	1016	(87.81)	141	(12.19)
Middle to high school	179 844	161 698	(89.91)	18 146	(10.09)	136 874	122 067	(89.18)	14 807	(10.82)
Above university level	578 128	525 328	(90.87)	52 800	(9.13)	480 277	431 031	(89.75)	49 246	(10.25)
Mother's occupation										
White collar	248 586	226 302	(91.04)	22 284	(8.96)	222 174	200 358	(90.18)	21 816	(9.82)
Pink collar	49 913	45 622	(91.40)	4291	(8.60)	55 892	50 352	(90.09)	5540	(9.91)
Blue collar	11 269	10 253	(90.98)	1016	(9.02)	34 162	30 778	(90.09)	3384	(9.91)
Other	449 765	406 244	(90)	43 521	(10)	306 080	272 626	(89)	33 454	(11)
Father's occupation										
White collar	460 665	418 441	(90.83)	42 224	(9.17)	308 080	277 357	(90.03)	30 723	(9.97)
Pink collar	129 453	117 547	(90.80)	11 906	(9.20)	74 722	67 339	(90.12)	7383	(9.88)
Blue collar	127 064	114 418	(90.05)	12 646	(9.95)	143 751	128 663	(89.50)	15 088	(10.50)
Other	42 351	38 015	(89.76)	4336	(10.24)	91 755	80 755	(88.01)	11 000	(11.99)
Medically underserved area										
Served area	746 440	676 596	(90.64)	69 844	(9.36)	607 027	544 011	(89.62)	63 016	(10.38)
Underserved area	13 093	11 825	(90.32)	1268	(9.68)	11 281	10 103	(89.56)	1178	(10.44)
Multiple birth										
No	729 897	681 366	(93.35)	48 531	(6.65)	591 309	548 038	(92.68)	43 271	(7.32)
Yes	29 636	22 581	(76.19)	7055	(23.81)	26 999	20 923	(77.50)	6076	(22.50)
First delivery										
Primiparity	399 692	365 404	(91.42)	34 288	(8.58)	328 770	297 260	(90.42)	31 510	(9.58)
Multiparity	359 841	323 017	(89.77)	36 824	(10.23)	289 538	256 854	(88.71)	32 684	(11.29)
Place of delivery										
Home	2626	2365	(90.06)	261	(9.94)	1780	1521	(85.45)	259	(14.55)
Hospital	755 048	684 305	(90.63)	70 743	(9.37)	615 713	551 885	(89.63)	63 828	(10.37)
Other	1859	1751	(94.19)	108	(5.81)	815	708	(86.87)	107	(13.13)
Mother's nationality										
Korean	729 476	661 090	(90.63)	68 386	(9.37)	589 995	529 013	89.66)	60 982	(10.34)
Other	30 057	27 331	(90.93)	2726	(9.07)	28 313	25 101	88.66)	3212	(11.34)
Father's nationality										
Korean	750 681	680 474	(90.65)	70 207	(9.35)	609 916	547 145	89.71)	62 771	(10.29)
Other	8852	7947	(89.78)	905	(10.22)	8392	6969	83.04)	1423	(16.96)

^a^ Data are presented as numbers and (percentage).

Table 2 presents the results of the segmented regression analysis used to assess the probability of a high‐risk newborn delivery after adjusting for all covariates. The likelihood of having premature infants or low‐birth‐weight deliveries after the establishment of reimbursement rates related to MFICU was estimated to a 0.4% decrease compared with that before the establishment of reimbursement rates (estimate 0.9599; 95% confidence interval [CI] 0.9449–0.9949; *P* = 0.0188). The likelihood of high‐risk newborn delivery during the pre‐intervention period showed an increasing trend (estimate 1.008; 95% CI 1.004–1.009; *P* < 0.001) but changed to a decreasing trend after the intervention (estimate 0.995; 95% CI 0.994–0.998; *P* = 0.003).

**TABLE 2:** Parameter estimate, standard errors and *P* values from the segmented regression models predicting the outcomes.

Parameter	Exp(*β*)	Exp(SE(*β*))	95% CI		*P* value
Risk of high‐risk newborn delivery					
Intercept *β*0	0.10	1.11	0.08	0.12	<0.001
Baseline trend *β*1	1.01	1.00	1.00	1.01	<0.001
Level change *β*2	0.96	1.01	0.94	0.99	0.019
Trend change *β*3	1.00	1.00	0.99	1.00	0.000
Follow‐up outcome trend *β*1 + *β*3	1.00	1.02	1.00	1.01	<0.001

Abbreviations: CI, confidence interval; SE, standard error.

**FIGURE 1:**
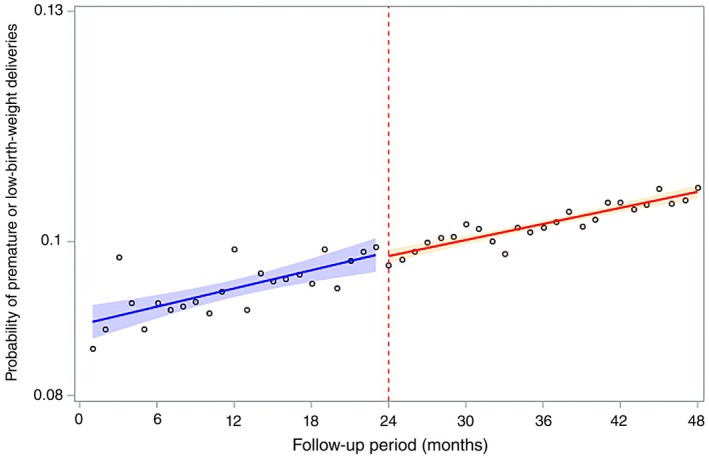
Factors associated with trends of high‐risk newborn delivery.

Table 2 presents the differences in the level and trend changes by calculating the parameter estimates. Figure 1 shows the outcome trends after the intervention. The probability of high‐risk births increased during the pre‐intervention period. At the time of introduction, the intervention showed noticeable interruptions and level changes.

We assumed that the risk of premature birth or low birth weight would vary according to sociodemographic characteristics; therefore, we performed subgroup analyses stratified by medically served area, legitimate child status, and multiple birth status. As shown in Table 3, the difference in level change at intervention, was more pronounced for those legitimate child (exp [*β2*] = 0.969; *P* = 0.02). In addition, those who delivered multiple births were associated with a larger difference in immediate effect size due to established MFICU reimbursement rates (Multiple births: exp. [*β2*] = 0.909; *P* = 0.02; Singleton birth: exp. [*β*2] = 0.977; *P* = 0.09).

**TABLE 3:** Subgroup analysis stratified by independent variables^a^.

	Risk of delivering a high‐risk newborn			
	Intercept Exp(*β*0)	Baseline trend Exp(*β*1)	Level change Exp(*β*2)	Trend change Exp(*β*3)
Legitimate child				
No	0.39	1.00	1.00	1.01
	(0.005)	(0.525)	(0.999)	(0.292)
Yes	0.09	1.01	0.97	1.00
	(<0.001)	(<0.001)	(0.020)	(0.000)
Multiple birth				
No	0.09	1.01	0.98	1.00
	(<0.001)	(<0.001)	(0.096)	(0.000)
Yes	2.07	1.01	0.91	1.00
	(0.098)	(<0.001)	(0.020)	(0.603)
Medically underserved area				
Served area	0.09	1.01	0.97	1.00
	(<0.001)	(<0.001)	(0.029)	(0.000)
Underserved area	0.07	1.01	0.87	1.00
	(<0.001)	(0.083)	(0.157)	(0.793)
Sex				
Male	0.11	1.00	1.00	1.00
	(<0.001)	(<0.001)	(0.961)	(0.117)
Female	0.09	1.01	0.94	1.00
	(<0.001)	(<0.001)	(0.001)	(0.000)
Mother's age				
≤ 19	0.13	1.02	0.99	0.95
	(0.049)	(0.040)	(0.963)	(0.002)
20–29	0.10	1.01	0.94	0.99
	(<0.001)	(<0.001)	(0.021)	(0.010)
30–39	0.35	1.01	0.98	1.00
	(0.375)	(<0.001)	(0.115)	(0.012)
40–49	0.09	1.01	0.97	1.00
	(<0.001)	(<0.001)	(0.029)	(0.001)
50–59	0.10	1.01	0.97	1.00
	(<0.001)	(<0.001)	(0.022)	(0.001)
Father's age				
≤19	0.00	1.01	0.86	0.97
	(<0.001)	(0.499)	(0.699)	(0.288)
20–29	0.11	1.01	0.94	0.99
	(<0.001)	(<0.001)	(<0.001)	(<0.001)
30–39	0.10	1.01	0.97	1.00
	(<0.001)	(<0.001)	(0.139)	(0.005)
40–49	0.10	1.01	0.99	1.00
	(<0.001)	(<0.001)	(0.036)	(0.012)
50–59	0.05	1.00	1.01	0.99
	(<0.001)	(0.001)	(0.778)	(0.306)
Mother's education level				
Below elementary school	0.02	1.01	0.86	1.00
	(<0.001)	(0.612)	(0.523)	(0.979)
Middle to high school	0.15	1.01	0.97	1.00
	(<0.001)	(<0.001)	(0.337)	(0.169)
Above university level	0.16	1.01	0.97	1.00
	(<0.001)	(<0.001)	(0.033)	(0.001)
Father's education level				
Below elementary school	0.43	1.00	1.15	1.00
	(0.302)	(0.721)	(0.591)	(0.982)
Middle to high school	0.14	1.01	0.97	0.99
	(<0.001)	(<0.001)	(0.219)	(0.002)
Above university level	0.07	1.01	0.97	1.00
	(0.020)	(0.604)	(0.972)	(0.593)
First delivery				
Primiparity	0.08	1.01	0.98	1.00
	(<0.001)	(<0.001)	(0.266)	(0.000)
Multiparity	0.13	1.01	0.96	1.00
	(<0.001)	(<0.001)	(0.021)	(0.030)
Place of delivery				
Home	0.09	1.01	1.93	0.97
	(<0.001)	(0.295)	(0.002)	(0.019)
Hospital	0.06	1.01	0.97	1.00
	(<0.001)	(<0.001)	(0.012)	(0.001)
Other	0.09	1.05	1.02	0.96
	(0.011)	(0.000)	(0.940)	(0.035)
Mother's nationality				
Korean	0.09	1.01	0.96	1.00
	(<0.001)	(<0.001)	(0.007)	(0.001)
Other	0.17	1.01	1.06	0.99
	(<0.001)	(0.003)	(0.303)	(0.119)
Father's nationality				
Korean	0.09	1.01	0.96	1.00
	(<0.001)	(<0.001)	(0.006)	(0.005)
Other	0.16	1.03	1.06	0.95
	(<0.001)	(<0.001)	(0.579)	(<0.001)
Mother's occupation				
White collar	0.04	1.00	0.97	1.00
	(<0.001)	(0.000)	(0.165)	(0.744)
Pink collar	0.14	1.01	0.93	0.99
	(<0.001)	(<0.001)	(0.151)	(0.001)
Blue collar	0.41	1.00	1.01	1.00
	(0.020)	(0.692)	(0.943)	(0.658)
Other	0.10	1.01	0.97	1.00
	(<0.001)	(<0.001)	(0.082)	(0.001)
Father's occupation				
White collar	0.08	1.00	0.97	1.00
	(<0.001)	(<0.001)	(0.059)	(0.841)
Pink collar	0.09	1.01	0.97	0.99
	(<0.001)	(<0.001)	(0.344)	(0.001)
Blue collar	0.14	1.01	0.93	1.00
	(<0.001)	(0.001)	(0.020)	(0.684)
Other	0.14	1.02	0.94	0.98
	(<0.001)	(<0.001)	(0.100)	(<0.001)

^a^Data are presented as Exp(*β*0) to Exp(*β*3) as indicated with *P* values in parenthesis.

Table 4 shows that when high‐risk births were defined separately as premature birth and low birth weight, the results indicated a more pronounced level change in preterm birth (estimate 0.953; 95% CI 0.925–0.980; *P* = 0.001). For low birth weight, no significant difference in level change was found, and both baseline trend and trend change values showed a slight increase similar to previous trends, followed by a slight decrease after the intervention. (Preterm birth: exp. [*β*1] = 1.004; *P* < 0.001; exp. [*β*3] = 0.997; *P* = 0.02; Low birth weight: exp. [*β*1] = 1.006; *P* < 0.001; exp. [*β*3] = 0.995; *P* < 0.001).

**TABLE 4:** Subgroup analysis stratified by high‐risk newborn baby.

Parameter	Exp(*β*)	Exp(SE(*β*))	95% CI		*P* value
Risk of preterm birth					
Intercept *β*0	0.06	1.10	0.05	0.07	<0.001
Baseline trend *β*1	1.00	1.00	1.00	1.01	<0.001
Level change *β*2	0.95	1.01	0.93	0.98	0.001
Trend change *β*3	1.00	1.00	1.00	1.00	0.020
Risk of low birth weight					
Intercept *β*0	0.06	1.10	0.05	0.07	<0.001
Baseline trend *β*1	1.01	1.00	1.01	1.01	<0.001
Level change *β*2	0.98	1.02	0.95	1.01	0.219
Trend change *β*3	1.00	1.00	0.99	1.00	<0.001

Abbreviations: CI, confidence interval; SE, standard error.

## 4 DISCUSSION

Our study analyzed the impact of an integrated care center for high‐risk pregnancies and neonates. We observed a significant decrease in the likelihood of premature or low‐birth‐weight deliveries after the intervention. As previous studies have pointed out, South Korea is currently facing a declining birth rate due to fewer marriages and later marriage ages.^18^, ^19^, ^20^, ^21^ Concerns are rising about the increasing age of pregnancy, which brings risks of complications during pregnancy and health issues in newborns.^18^ Adverse outcomes, such as premature birth and low‐birth‐weight infants, are significant societal concerns because of their association with higher infant mortality rates and increased medical costs.

Childbirth and maternity care are essential healthcare fields requiring appropriate government intervention.^22^ The infrastructure for treating high‐risk mothers and newborns constitutes a high‐cost, low‐profit sector in which voluntary private investment is inadequate. Therefore, proactive government support is crucial. In Korea, the Ministry of Health and Welfare aims to support healthy childbirth by establishing integrated and systematic maternity care systems for high‐risk mothers and newborns.^11^ This includes the introduction of relevant reimbursement rates to fund the development of specialized personnel, facilities, and equipment. Advanced economies are increasingly establishing and enhancing integrated maternity care systems and support mechanisms.^23^ Since 1996, the Japanese government has actively promoted integrated maternity center projects to operate as a comprehensive care system. This approach has contributed to Japan maintaining a low infant mortality rate of 1.9 deaths per 1000 births, compared with the OECD (Organization for Economic Co‐operation and Development) average of 4.2 deaths per 1000 births.^24^

The subgroup analyses further showed that the effects varied across sociodemographic characteristics. Specifically, the immediate impact was more pronounced among births classified as legitimate. For example, extramarital births carry a heightened risk of premature delivery before 37 weeks of pregnancy^25^, ^26^ and an increased likelihood of low‐birth‐weight neonates (weighing less than 2.5 kg).^27^ These conditions also increase the risk of fetal and neonatal mortality and the occurrence of sudden infant death syndrome.^28^ According to systematic reviews, the risk of premature birth in extramarital pregnancies is reported to be 1.22 times higher.^26^ In South Korea, societal attitudes towards extramarital births remain negative, leading to inadequate legal and institutional protection and more restrictive prenatal care services for unmarried pregnant women.^29^ Research has shown that unmarried women often experience delayed pregnancy awareness and receive lower levels of prenatal care, which are linked to negative pregnancy outcomes such as premature birth and low birth weight.^30^ Our study also found that extramarital births were associated with an increased risk of adverse pregnancy outcomes, including premature birth and low birth weight, with limited effectiveness of the interventions observed. Hence, there is a critical need to develop systematic service programs tailored to these groups.

In addition, the immediate benefits of MFICU expansion were more pronounced, especially for those living in underserved areas, those with low levels of multiple births, low maternal education, and a higher risk of premature births, such as multiple births or infants having low birth weights. Access to health care is important.^31^, ^32^ There was a significant decline in the previously revealed risk group for high‐risk births, suggesting that the expansion of integrated care centers may have helped with a systematic and efficient care system. In addition, the risk of high‐risk births tended to increase in non‐citizen parents, which could be the result of being excluded from health insurance benefits.

The present study has several limitations. First, the Statistics Korea population dynamics data on population trends that we analyzed included sociodemographic and birth information for the total births in South Korea; however, they lacked specific details about mothers or other medical service utilization. Therefore, we could not consider alternative causes or exclude residual confounding factors from the unmeasured variables. Additionally, we were unable to ascertain whether the mothers used the integrated MFICU because direct usage information was unavailable. Third, we could not control for other informal benefits and interventions provided to mothers and fetuses at the same time as the introduction of MFICU support. Hence, it is possible that these associations were overestimated. Finally, owing to the lack of specific information, such as the location where mothers gave birth or hospital details, we could not exclude potential confounding effects related to these factors. Future research incorporating such information will be necessary.

Nevertheless, the present study has several strengths. One of the major strengths is that it analyzed a large‐scale data set, including total births in South Korea, which allowed us to evaluate the relevance of policies and generalize our findings. Furthermore, we used 48 time points (24 months before and after the intervention) to capture and analyze temporal trends and changes over time more accurately.

In conclusion, the expansion of MFICU into obstetric care strategies has demonstrated significant benefits in reducing high‐risk births, such as premature births and low birth weight neonates. Adopting a multidisciplinary approach, conducting thorough risk assessments before conception, ensuring early admission for delivery, and providing continuous antenatal care are vital components of contemporary obstetric care strategies.^33^ These measures are crucial for reducing social and economic burdens, creating a healthy birth environment, and improving peripartum outcomes. Hence, to effectively care for high‐risk mothers and prevent high‐risk births, it is essential to enhance obstetric care levels through the efficient utilization of these units and the implementation of continuous medical support policies.

## AUTHOR CONTRIBUTIONS

YS developed the idea for the study and conducted the data collection and analysis, and wrote the initial manuscript. YS, SI, and EC contributed to the interpretation of the data and revision of the manuscript.

## CONFLICT OF INTEREST STATEMENT

The authors have no conflicts of interest.

## Data Availability

Data openly available in a public repository that issues datasets with DOIs The data that support the findings of this study are openly available in Statistics Korea at http://doi.org/10.23333/P.101003.001, reference number 13.
